# The polyamine transporter *Slc18b1(VPAT)* is important for both short and long time memory and for regulation of polyamine content in the brain

**DOI:** 10.1371/journal.pgen.1008455

**Published:** 2019-12-04

**Authors:** Robert Fredriksson, Smitha Sreedharan, Karin Nordenankar, Johan Alsiö, Frida A. Lindberg, Ashley Hutchinson, Anders Eriksson, Sahar Roshanbin, Diana M. Ciuculete, Anica Klockars, Aniruddha Todkar, Maria G. Hägglund, Sofie V. Hellsten, Viktoria Hindlycke, Åke Västermark, Ganna Shevchenko, Gaia Olivo, Cheng K, Klas Kullander, Ali Moazzami, Jonas Bergquist, Pawel K. Olszewski, Helgi B. Schiöth

**Affiliations:** 1 Department of Pharmaceutical Biosciences, Uppsala University, Uppsala, Sweden; 2 Department of Neuroscience, Functional Pharmacology, Uppsala University, Uppsala, Sweden; 3 Faculty of Science and Engineering, University of Waikato, Hamilton, New Zealand; 4 Department of Chemistry, Uppsala University, Uppsala, Sweden; 5 Department of Molecular Sciences, Swedish University of Agricultural Sciences, Uppsala, Sweden; 6 Institute for Translational Medicine and Biotechnology, Sechenov First Moscow State Medical University, Moscow, Russia; Stanford University School of Medicine, UNITED STATES

## Abstract

SLC18B1 is a sister gene to the vesicular monoamine and acetylcholine transporters, and the only known polyamine transporter, with unknown physiological role. We reveal that *Slc18b1* knock out mice has significantly reduced polyamine content in the brain providing the first evidence that *Slc18b1* is functionally required for regulating polyamine levels. We found that this mouse has impaired short and long term memory in novel object recognition, radial arm maze and self-administration paradigms. We also show that *Slc18b1* KO mice have altered expression of genes involved in Long Term Potentiation, plasticity, calcium signalling and synaptic functions and that expression of components of GABA and glutamate signalling are changed. We further observe a partial resistance to diazepam, manifested as significantly lowered reduction in locomotion after diazepam treatment. We suggest that removal of *Slc18b1* leads to reduction of polyamine contents in neurons, resulting in reduced GABA signalling due to long-term reduction in glutamatergic signalling.

## Author summary

A fundamental function of the nervous system is its ability to modulate and change the connections between nerve cells, and this forms the basis for memory and learning. This is most well studied for synapses that are using the neurotransmitter glutamate, and a central part of this is referred to Long Term Potentiation. This process is dependent on a specific glutamate receptor called the NMDA receptor, and the function of this receptor can be controlled by various mechanisms. Here, we show that polyamines can regulate this receptor and that lack of polyamines result in impaired learning and memory. Polyamines are small peptides made by many different cells in the body, including cells in the brain, and by removing a gene coding for a transporter important for the release of polyamines in nerve cells of mice, we show that polyamines are important for proper function of the glutamate system. We also show the deletion of this gene result in fundamentally rearranged GABA and glutamate systems, resulting in the mice having a much higher tolerance for the sedative drug benzodiazepines. Polyamines and targets for these molecules could be important points of intervention for future drugs aiming at modulating the glutamatergic system.

## Introduction

Polyamines (PAs) are endogenous compounds and the most common PAs produced by mammalian cells are spermidine (Spd), spermine (Spm) and putrescine [1]. The polyamines are present in all living cells and are essential for normal cell function, cellular growth and differentiation [2]. Spd and Spm are produced by mammalian neurons from arginine and methionine via the rate limiting enzyme ornithine decarboxylase (ODC) [3], which is essential for embryonic development [4]. They are stored in synaptic vesicles and co-released with neurotransmitters upon depolarization and have been shown to act as neuromodulators. At low concentrations extracellular polyamines potentiate [5] the NMDA receptor and at high concentrations they act as blockers on the same receptor [6], by occupying specific binding sites. The potentiation of the NMDA receptor has been shown to, at the physiological level, result in enhanced memory performance [7] and plasticity [8]. The polyamines can also potentiate the kinate receptor and block the AMPA receptor upon binding to their specific sites [9].

The mechanism of storage and transport for PAs was for a long time a mystery and most of the details regarding this are still unknown. Recently it was suggested that the solute carrier (SLC) SLC18B1 was able to transport polyamines *in vitro* using synthetic liposomes. It was suggested that SLC18B1 codes for a vesicular transporter and hence named vesicular polyamine transporter (VPAT)[10]. These data were however obtained only from *in vitro* experiments in synthetic liposomes and although the study clearly suggested that SLC18B1 have transport ability for polyamines, it did not show if this transport is also relevant *in vivo* nor did it show any physiological relevance of this transport.

The SLC18 family contains four members in total, two vesicular monoamine transporters VMAT 1 (SLC18A1) and 2 (SLC18A2) and the vesicular acetylcholine transporter (VACHT, SLC18A3). SLC18A2 is found in all neurons which signal through any of the mono amines or through serotonin in the PNS and CNS, and is the only protein capable of transporting these transmitters into synaptic vesicles for further release and is hence crucial for all monoaminergic signalling. VMAT1 is found in neuroendocrine cells and has the same function as VMAT2 has in neurons [11]. Similarly, VACHT is responsible for transporting acetylcholine into synaptic vesicles [11], and is necessary for cholinergic signalling in adults [12]. We have previously shown that SLC18B1 is a phylogenetically distant member of the SLC18 family with widespread expression in the brain [13].

In this paper we present the first transgenic mice where SLC18B1 has been removed. We show that removal of SLC18B1 results in markedly lower concentrations of polyamines in the brain. We performed thorough behavioural characterization of the KO mouse and found clear evidence for effects on memory while many other behavioural functions remained intact. Expression and proteomics data suggest influence on genes and proteins related to Long Term Potentiation (LTP) and plasticity, calcium signalling and synaptic functions delineating plausible mechanisms for the behavioural effects.

## Results

### 1. Generation and verification of the Slc18b1 knockout

**Fig 1:**
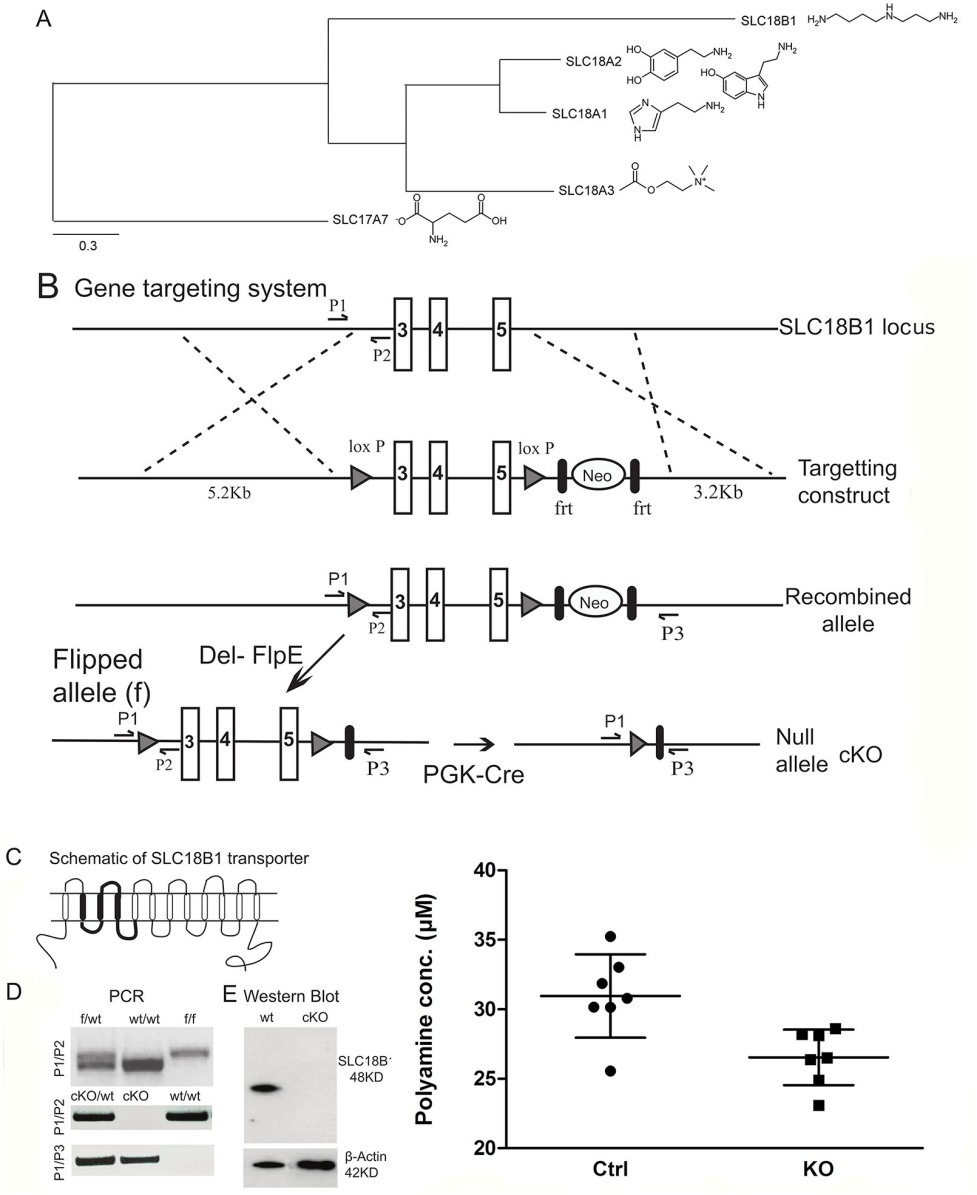
Gene targeting of the Slc18b1 locus results in specific loss of SLC18B1 protein expression. A) Phylogenetic tree showing the phylogenetic relationship of the proteins within the SLC18 family. SLC18A7 (vGluT1) is used as outgroup. The figure includes schematic images of the main substrates of transport for each transporter. B) The gene targeting strategy shows exons 3, 4 and 5 in the Slc18b1 locus flanked by 5’and 3’ loxP site followed by neo cassette flanked by frt sites resulting in recombinant allele (f). The locations of the PCR primers used in the screening are labelled P1, P2 and P3. The flipped allele is produced by crossing the heterozygous floxed mice with the deleter; Del-FlpE mice. The flipped mice are further crossed with PGK-cre mice to generate Slc18b1 null mutant mice. C) A schematic view of the Slc18b1 transporter with 12 transmembrane domains. The targeted region corresponds to the transmembrane domain 2, 3 and 4, and loops 2, 3 and 4. D) The PCR screen of the flipped and the null mutant mice with wild type mice and heterozygous mice using the primers illustrated in A. E) Western blot to detect the Slc18b1 protein in ctrl and knock out brain homogenate. The 48KD band in the ctrl mice corresponds to the Slc18b1 protein and β- actin was used as loading control. F) Measurement of total polyamine content in brain of cKO and ctrl mice. cKO has significantly (P = 0.011) lower total polyamine content in brain compared to ctrl.

SLC18B1 is a member of the SLC18 family, which is most closely related to the SLC17 family [14]. SLC18B1 has been shown to transport spermidine and other polyamines [10] while the other members of the SLC18 family are vesicular monoamine (SLC18A1 and SLC18A2) and vesicular acetylcholine transporters (SLC18A3) (Fig 1A). We generated a *Slc18b1* transgenic allele by replacing part of the *Slc18b1* gene with a targeting construct by homologous recombination in ES cells (Fig 1B). Successfully targeting produced a modified allele with a loxP site preceding exon 3, 4 and 5, coding for the putative transmembrane regions 2, 3 and 4 (Fig 1C) and a neomycin selection cassette flanked by Frt sites, followed by a second loxP site. We confirmed the correct targeting event in the ES cells and in the animals by a PCR strategy (Fig 1D). The neo cassette was removed by crossing *Slc18b1*^*f*/+^ mice to Deleter-FlpE mice [15] and the flipped *Slc18b1*^*f/f*^ were viable and fertile and subsequently crossed to PGK-Cre mice [16] to delete the targeted region and generate null mutant mice, *S lc18b1*^*f/f;PGK-Cre*^ (cKO), the genotype of these mice were verified using a PCR assay (Fig 1D). We performed western blot on homogenate from brain tissue from both control (ctrl) and cKO mice to detect the SLC18B1 protein. We could detect the SLC18B1 protein in the ctrl homogenate but the band was completely absent in the cKO homogenate (Fig 1E). This shows that deletion of the targeted region results in the complete absence of SLC18B1 protein product in null mutant mice.

### 2. Polyamine levels in neurons

Next we investigated the levels of polyamines of ctrl and cKO mice (Fig 1F). Brain homogenate were analysed for polyamine content using a enzymatically based polyamine quantification kit. We found that polyamine levels were significantly lower (P = 0.011) in cKO compared to ctrl mice in a Mann Whitney U test. This shows that Slc18b1 is functionally relevant in regulating total polyamine levels in the brain.

### 3. Primary behavioural analysis of the KO mouse

**Fig 2:**
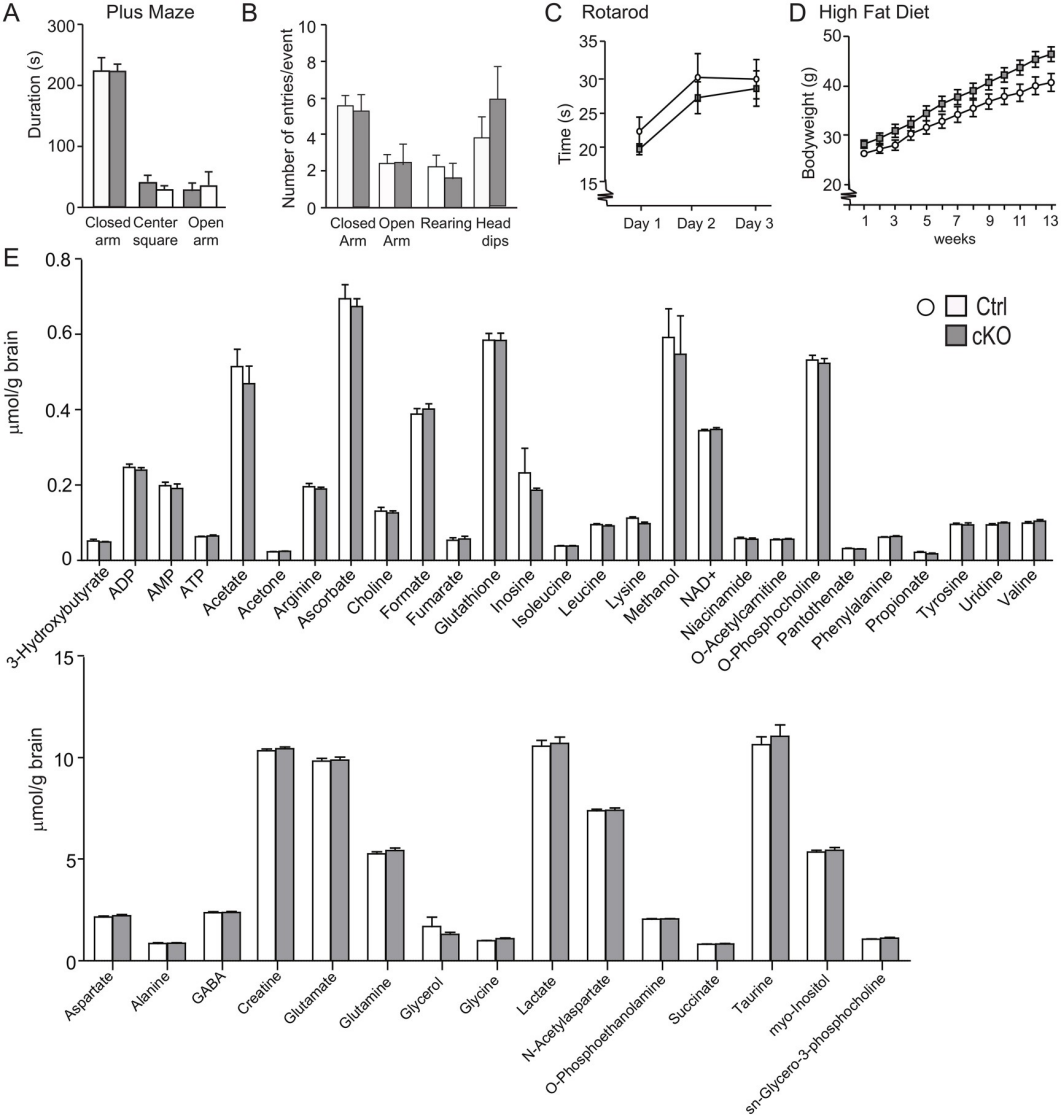
The cKO mice are not hyperactive and do not displays an anxiety like phenotype. A) Elevated plus maze analysis on adult male cKO mice and ctrl littermates. The cKO mice spent the same amount of time in the in the four arms as compared to ctrl mice. B) The entries in the closed and open arm are not significantly altered in the cKO mice as well as rearing and head dips (cKO male mice, n = 11; ctrl male mice, n = 9). C) No significantly difference in motor performance were observed between cKO mice and ctrl in a rotarod apparatus for three trail/day over three consecutive days (n = 10/genotype) D) No significantly changes in bodyweight over a period of 13 weeks when cKO and ctrl mice where given a high-fat/high-sugar diet available ad libitum (n = 12/genotype) E) Levels of 42 different metabolites in NMR but there were no significantly differences between cKO and ctrl group.

3a. Motor functions: We further analysed the cKO and ctrl mice in several behavioural paradigms. In the elevated plus maze (Fig 2A), we found no difference between the genotypes in the preference of open or closed arms, nor in the preference for the centre square (Fig 2A). We also found no difference in rearing and number of head dips measured in the elevated plus maze (Fig 2B). We interpret these data as that there is no anxiety phenotype in the transgenic line. We further tested their motor function in the rotarod setup (Fig 2C) and also here we found no difference between the two lines. To investigate if there was a growth phenotype we fed mice high caloric food and monitored their weight gain over 13 weeks. We found that the cKO did not gain significantly more weight at the end of this period (Fig 2D) although there was a trend pointing towards cKO being heavier. We further examined 42 different metabolites in brain homogenates from cKO and ctrl mice using NMR (Fig 2E) and found no significantly differences between cKO and ctrl group. Taken together this data suggest that there is neither a growth phenotype, nor any major metabolic phenotype. Also, we found no phenotype regarding basal behaviour from deletion of the *Slc18b1* gene.

**Fig 3:**
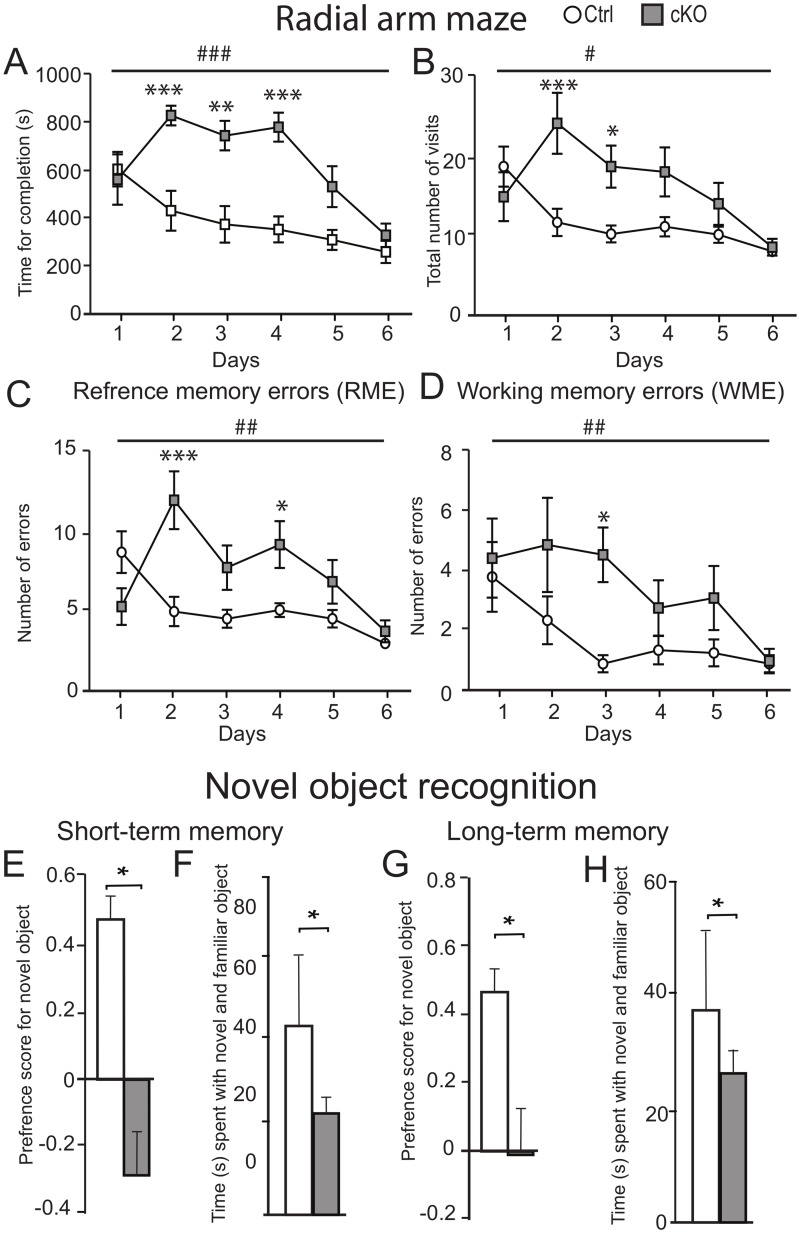
Impaired memory function in cKO mice compared to ctrl. A-B) Radial arm maze was used to evaluate memory for a 6 day trail. The cKO mice took significantly longer time to complete the task of retaining four pellets (A) as well as made over all more visits in all arms of the maze B). The reference memory (C) and the working memory (D) are significantly worse in the cKO mice as compared to ctrl mice (n = 10/genotype). Significant main genotype effect observed by two way Anova analysis are illustrated by #p<0.05, ##p<0.01 and ###p<0.001; differences by Bonferroni post hoc test are shown by *p<0.05, **p<0.01 and ***p<0.001 E-H) Recognition memory function was assessed in a three day protocol consisting of a habitation day (day one), test for short term memory (day 2) and long-term memory (day 3) (n = 5/genotype). On day 2 (short-term memory), the cKO mice displayed a significantly less preference for the novel object (Mann Whitney U-test p<0.05) (E), and the cKO mice spent a significantly lower time with the novel object (Mann Whitney U-test p<0.05) (F). On day 3 (long-term memory), the ctrl mice displayed a significantly strong object preference for the novel object (G) (Mann Whitney U-test p<0.05) and the cKO mice spent significantly shorter time with the novel object (Mann Whitney U-test p<0.05) (H). Data represent mean ±SEM.

3b. Memory functions: The KO animals were subsequently subjected to two behavioural paradigms for memory performance. The first test was the eight arm radial maze, a spatial working-memory task considered to be largely dependent on hippocampal function [17]. The test was performed for six subsequent days in order to see how the time to consume the pellets as well as the number of errors performed decreased with each day of testing (Fig 3C–3F). On the first trial day, both genotypes took a similar amount of time to complete the task (ctrl mean = 604.8±72.45 s, cKO mean = 561.33±105.5 s, Mann Whitney U test p>0.05). With each subsequent day of testing, ctrl mice took less time to complete the task while the cKO mice did not improve, showing a significantly impaired learning ability (two way repeated measure ANOVA, Genotype F(_1,18_) = 34.55, p<0,0001; Bonferroni posttest day 2, p<0,001; Bonferroni posttest day 3, p<0,01, Bonferroni posttest day 4, p<0,001) (Fig 3C). The cKO mice made significantly more visits to all eight arms compared to ctrl mice (two way repeated measure ANOVA, Genotype F(_1,18_) = 7.46, p = 0,037; Bonferroni posttest day 2, p<0,001; Bonferroni posttest day 3, p<0,05) (Fig 3D). We investigated the working memory error (WME), which was defined as re-entry in to a previously baited (now empty) arm, the cKO mice performed significantly more WMEs, (two way repeated measure ANOVA, Genotype F(_1,18_) = 8.93, p = 0.0065; Bonferroni posttest day 3, p<0,05) (Fig 3F). We also analysed spatial memory in reference memory error (RME), defined as entries in to a never baited arm. The cKO performed significantly more RMEs (two way repeated measure ANOVA, Genotype F(_1,18_) = 9.48, p = 0,037; Bonferroni posttest day 2, p<0,001; Bonferroni posttest day 4, p<0,05) (Fig 3E). These results suggest that the cKO mice have impairments in spatial working-memory and/or hippocampal function. We also tested recognition memory using the novel object recognition setup. On day 1 (habituation day), each mouse was habituated to an arena with identical objects placed at each end for 10 minutes. On day 2 (short-term memory), the ctrl mice displayed a significantly stronger preference for the novel object (Mann Whitney U-test p<0.05) (Fig 3G), and the cKO mice spent a significantly less time with the novel object (Mann Whitney U-test p<0.05) (Fig 3H). These data suggest that the KO mice have deficits in short-term recognition memory and/or hippocampal function. On day 3 (long-term memory), the ctrl mice once again displayed a significantly strong object preference for the novel object (Fig 3I) (Mann Whitney U-test p<0.05) and the cKO mice spent significantly shorter time with the novel object (Mann Whitney U-test p<0.05) (Fig 3J). In addition to deficits in short-term memory, these data also suggest that the cKO mice have deficits in long-term memory.

**Fig 4:**
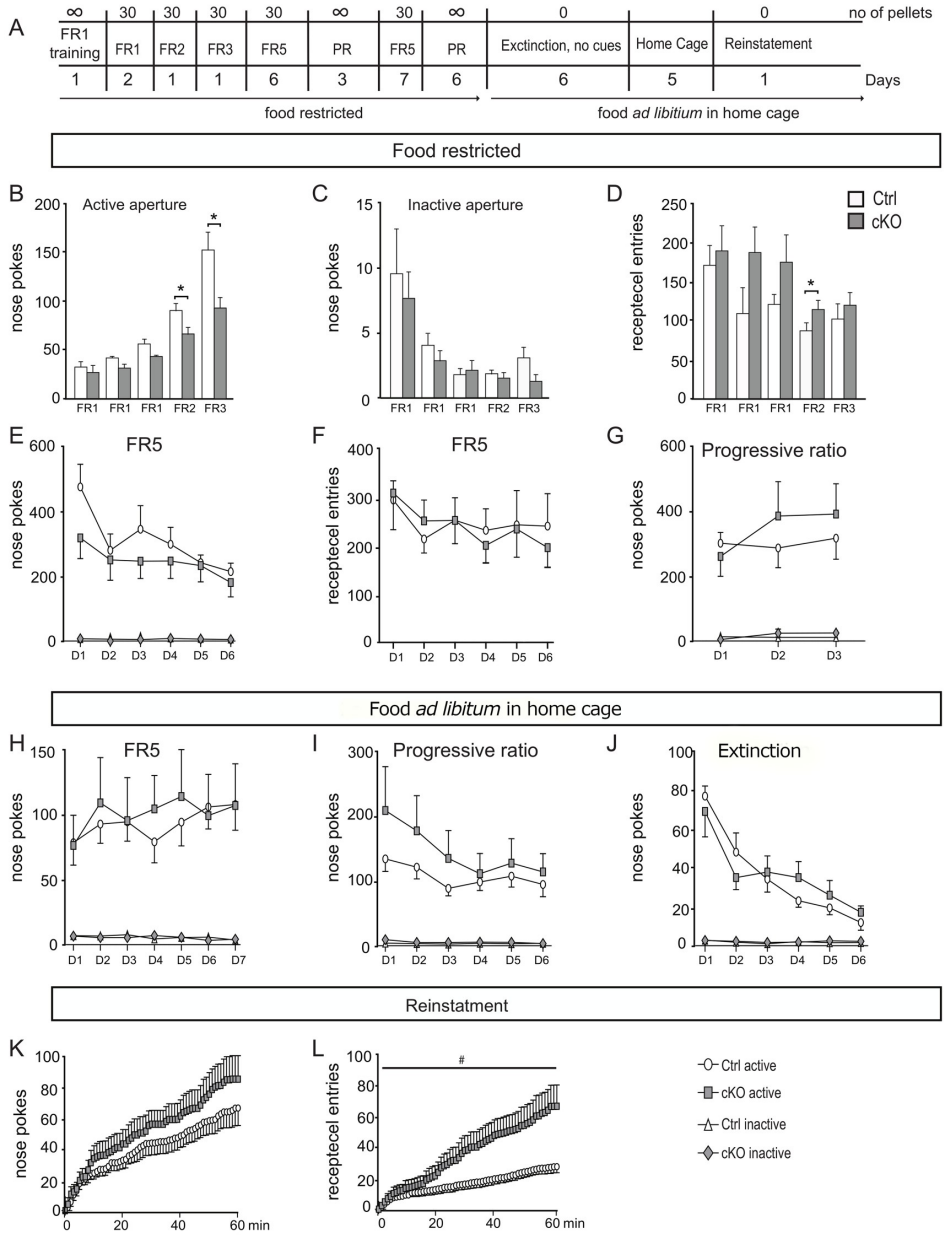
The cKO mice make more entries in the food receptacle during reinstatement. A) The mice (cKO male mice, n = 7; ctrl male mice, n = 8) were analysed for self-administration of sucrose in the operand setting with two feeders, one of which delivers pellets upon head entry (active aperture) and the other did not (inactive apparatus). When a mouse made a head entry at the active feeder, a sugar reward was delivered, and simultunasly, light and sound cues were presented to confirm the chooise; a head entry in the inactive feeder neither produced a reward nor a light or cue. B) Mice were trained to nosepoke on a fixed ratio 1(FR1) schedule for sucrose pellets during mild food restriction for three days with a max of 30 sucrose pellets, the cKO mice nose poke equally number as the ctrl mice. On the FR2 and FR3 schedule the cKO mice nose pokes significantly lower number as compared to ctrl (Mann Whitney U-test p = 0.037 and p = 0.0397). C) There were a clear decrease of nose pokes in the inactive apparatus for both cKO and ctrl mice during the FR1-FR3 schedule. D) The cKO mice made more receptacle entries overall during the FR1-FR3 schedule but significantly more on the FR2 schedule (Mann Whitney U-test p = 0.0371). E) The number of nose pokes in the active and inactive hole was not significantly altered in the cKO mice as well as the number of receptacle entries (F). G)The progressive ratio(PR) was performed over a three day period and showed no alteration in the cKO mice. H) During a 7 day trail on FR5 paradigm the genotypes performed equally, both on active and inactive nose poke hole I) During PR, no difference was seen in head entries in the active or inactive nose poke hole. J-L) Cognitive ability testing. During reinstatement (K-L), the mice were presented to the original task after an extinction period (J). J) For six consecutive days, the active feeder delivered no light, sound or pellet (extinction). For both groups, the amount of head entries strongly decreases. K-L) during the reinstatement the active feeder delivered both light and sound cues, but no sugar pellets. The numbers of nose pokes are not different in the cKO mice (K) but the cKO mice make significantly more receptacle entries as compared to ctrl two way repeated measure ANOVA, Genotype F(1,12) = 5.57, p = 0,0345 (L). Data represent mean ±SEM.

3c. Operant self administration: Further, we used operand chambers to study voluntary consumption of a rewarding substance. The mice were analysed for self-administration of sucrose in the operand setting (Fig 4A). To determine whether acquisition of an operand task in the cKO mice were impaired, mice were first trained to nosepoke on a Fixed Ratio 1(FR1) schedule for sucrose pellets during mild food restriction. The number of nose pokes during the FR1 schedule was not significantly different between cKO and ctrl mice (Mann Whitney U-test p>0.05). However, the ctrl mice nose poked significantly more during FR2 (Mann Whitney U-test p = 0.037) and FR3 (Mann Whitney U-test p = 0.0397) schedule. There were no difference in number of nose pokes in the inactive apparatus; this shows that the cKO mice had learnt the goal-directed response (Fig 4C). The cKO mice did more receptacles entries in all trainings schedules, but significantly more entries on the FR2 schedule (Mann Whitney U-test p = 0.0371) (Fig 4D). The cKO mice did not self-administrate more sucrose pellets during the FR5 schedule (Fig 4E), nor did they enter the food receptacle in a different fashion compared to ctrl mice (Fig 4F). After the ctrl and cKO mice had established a biased response in the active nosepoke apparatus, the reinforcement schedule was changed to progressive ratio (PR) to further determine the reinforcing effects of sucrose (Fig 4G). The PR paradigm has been described as a measure for the motivational aspect of consumption as compared to the FR5 schedule which is used to measure consumption rate [18]. There was no significant effect of genotype on the active nose poke hole. The average breakpoint (the number of nose pokes made to obtain the last reinforcement of the session) was not significantly different between ctrl (11.79±0.65) and cKO mice (12.79±0.77) (Mann Whitney U test p>0.05). Inactive responses were low during all reinforce/ schedule conditions and did not differ between the two genotypes (Fig 4C, 4E–4J).

3d. Feeding and growth: Since self-administration of sucrose pellets during mild food restriction could indicate a generalized increased in consumption responding to any food or reward (hunger driven feeding), we allowed the mice free access to rodent chow in their home cage to investigate sucrose reward-driven feeding. No significant differences were observed between the groups during FR5 and PR schedule in the reward-driven feeding. Lastly the mice were subjected to an extinction -reinstatement phase. There was no significant difference between cKO mice and ctrl mice in the number of nosepokes during the extinction phase. The reinstatement was one single session five days after the final extinction day, where the mice were on a schedule in which responding at the active nose poke apparatus produced both light and sound, but no pellet delivery. The cKO mice did not differ in the number of nose pokes, but they entered the food receptacle significantly more times as compared to ctrl mice (two way repeated measure ANOVA, Genotype F(_1,12_) = 5.57, p = 0,0345). Analysis of operant sugar consumption behaviour thus demonstrates that the cKO mice displayed normal reward-related learning, motivation, and ability for task-switching and their consummator of sugar eatables was not changed. However the cKO mice ability to learn the task in reinstatement was significantly impaired indicating that the cKO mice had an impaired memory function.

### 4. Expression arrays

**Fig 5:**
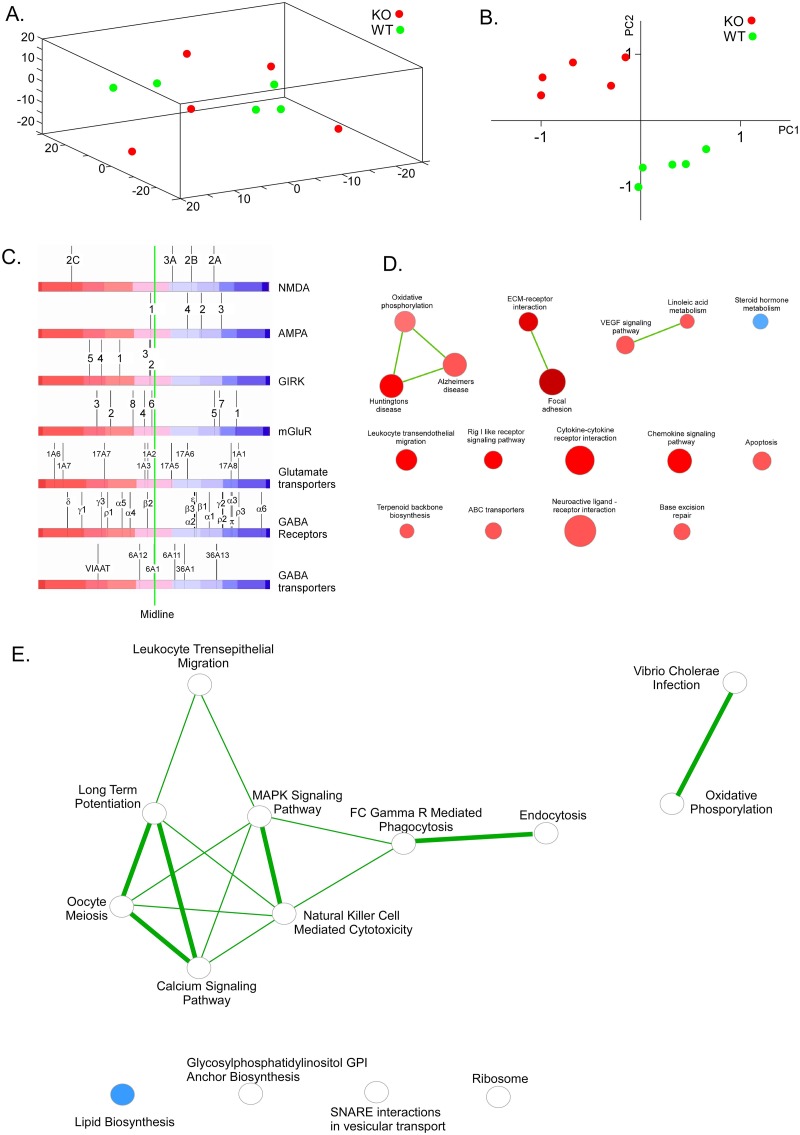
Clear differences in global gene expression in the adult brain in the cKO mice compared to ctrl mice. A) PCA plot in three dimensions showing expression values, including all 28794 genes from the Affymetrix microarray, for 5 cKO and 5 ctrl mice. B) PCA plot in two dimensions including the 500 genes with most significant differential expression between the two lines. The two vectors plotted explained 98.5% of the variation in the dataset. C) Gene Set Enrichment Analysis of the cKO and ctrl mice arrays. Each black vertical line represents one gene, and the position on the red to blue scale bar represents the average expression of the gene. Therefore, any line positioned to the left of the midline indicates an up regulation in the cKO animals compared to the ctrl and any line positioned to the right of the midline indicates down regulation in the knockout. D) KEGG pathway analysis on the 500 most changed transcript in the cKO adult mice brain, and 13 pathways are significantly altered. E) Mass spectrometry on the same brains as the expression array. The hydrophobic membrane protein and the water soluble protein fraction showed that 9 and 25 proteins (total 34 proteins), in respectively fraction, were significantly changed between cKO and ctrl mice. The KEGG pathway analysis was performed on the 34 changed proteins and 14 pathways were identified as significantly changed between cKO and ctrl mice.

Next, we performed expression micro arrays on cKO and ctrl mice to investigate differences in global gene expression in the adult brain. Expression data for 28794 transcripts were obtained for each genotype. In a PCA analysis we saw no clustering in three components that were dependant on experimental conditions and we saw no clustering based on genotype (Fig 5A). We therefore preformed a PCA analysis based the 500 transcripts with lowest P-value, adjusted for multiple comparisons using the false discovery rate method [19, 20], for differential expression. Here we saw a clear clustering based on genotype in two PCA components, which together explained 98.5% of the variation in the data, (Fig 5B). This suggests that there are relevant differences between the lines considering the most differentially expressed genes. We subsequently performed a Gene Set Enrichment Analysis (GSEA) [21, 22] for a number of gene sets involved in GABAergic and glutamatergic signalling (Fig 5C). We saw differential expression among these genes that can be explained by altered excitatory and / or inhibitory signalling, see the Discussion section. We used KEGG [22] to perform pathway analysis on the same 500 hundred most change transcripts as used for two component PCA analysis and found significant enrichments in 13 different pathways (Fig 5D) in the cKO mice compared to ctrl mice. Most of these pathways are involved in neuronal signalling, the immune system and hormone biosynthesis.

### 5. Proteomics

Next, we performed proteomics analysis using mass spectrometry on the other hemisphere of the brains used for expression microarrays. For this we analysed the hydrophobic membrane protein fraction separately from the water soluble proteins to increase the number of detected proteins. We found that 9 and 25 (a total of 34) proteins were significantly changed between cKO and ctrl mice in the respective fractions. Out of these 34 proteins, transcripts for 22 were also changed in the expression microarray in the same direction. We applied KEGG pathway analysis on the 34 changed proteins [23] and identified 14 pathways which were statistically significant enriched using the entire genome as reference set (Table 1, Fig 5E). Similar to what was seen for the RNA expression analysis, pathways involved in neuronal signalling and specifically LTP, the immune system and biosynthesis were identified as significantly enriched.

**Table 1:** Significantly differentially expressed proteins from table one and their associated KEGG pathways.

KEGG pathway	Proteins
Phagasome	Tuba4a, Tuba1a, Tubb5, Atp6v1c1, Atp6v1b2, Tubb2b
Gap junction	Tuba4a, Tuba1a, Tubb5, Tubb2b
Gastric acid secretion	Atp1a3, Ezr, Camk2a
Long-term potentiation	Ppp3cb, Camk2a, Rap1a
Collecting duct acid secretion	Atp6v1c1, Atp6v1b2
Leukocyte transendothelial migration	Ezr, Rhoa, Rap1a
Regulation of actin cytoskeleton	Ezr, Rhoa, Pak1, Pip4k2a
Vasopressin-regulated water reabsorption	Vamp2, Aqp4
T cell receptor signaling pathway	Ppp3cb, Rhoa, Pak1
Axon guidance	Ppp3cb, Rhoa, Pak1
Neurotrophin signaling pathway	Rhoa, Camk2a, Rap1a

### 6. Follow up behavioural testing

**Fig 6:**
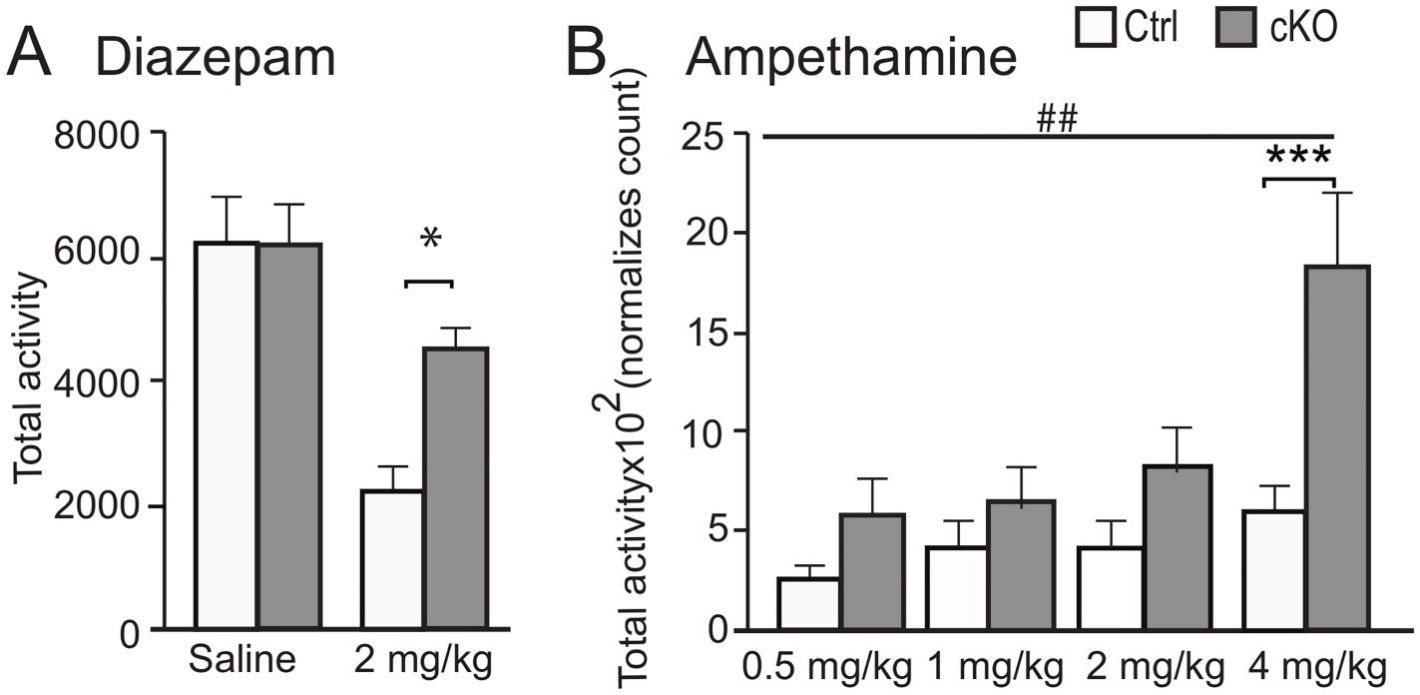
Increased sensitivity to amphetamine and decreased sensitivity diazepam cKO mice. A) Measurements of total activity over a period of 60 minutes in an automated activity chamber. There is no difference between cKO and ctrl mice (left set of bars) when injected i.p with 10 ml/kg saline, while there is a clear significant difference (Mann-Whitney U-test *p = 0.003) between cKO and ctrl mice treated with 2 ml/kg diazepam (cKO male mice, n = 16; ctrl male mice, n = 9). B) Mice were injected with 10 ml/kg saline and after a wash out period the mice were subjected to four doses of amphetamine in a scrambled fashion. The locomotion data for each animal was then normalized against its own saline measurements. The cKO mice displayed an increased sensitivity for amphetamine on all doses as compared to ctrl mice (n = 10/genotype).

Following the findings from the transcriptomics and proteomics study, we investigated if these molecular changes in the brain did also affect the behaviour of the animals by performing a series of behavioural experiments to specifically target GABA and glutamatergic signalling and LTP. We investigated their locomotor behaviour in automated locoboxes over 60 min. When we administered i.p. 10 ml/kg saline to cKO and ctrl mice there were no differences in locomotion between genotypes (Fig 6A). However, we found that when injected with i.p. 2 ml/kg diazepam, a benzodiazepine functioning as a positive allosteric modulator for GABA, the cKO showed a significant (Mann Whitney t-test p = 0.003) lower reduction in total activity compared to ctrl mice (Fig 6A). This is interesting, as it shows that the cKO mice have a partial resistance to the effect of benzodiazepines. We subsequently did a similar experiment with amphetamine, although here each animal where subjected to the test under the influence of first saline and then, after a washout period, under influence of amphetamine at different doses in a scramble fashion. The locomotion data for each animal was then normalized against its own saline measurements. Here we found that the cKO mice were more sensitive to the effect of amphetamine than ctrl mice (two way repeated measure ANOVA, Genotype F(_1,22_) = 14.1, p = 0,0011; Bonferroni posttest 4 mg/kg, p<0,001) (Fig 6B).

### 7. Analysis of genetic variants in the human SLC18B1 gene

Six independent variants were nominally associated with memory scores. The strongest signal was identified at rs11962883 (p_raw_ = 0.016), which was in LD with rs10484629 (r^2^ = 0.97, D’ = 0.99). **TMT A**. TMT A scores were significantly associated with two variants (rs531880979, p_raw_ = 0.030, and rs548918202, p_raw_ = 0.037). The former one is located within 3’ UTR of the *SLC18B1* gene. **TMT B**. Six variants had been associated with TMT B scores. Two of the identified SNPs were in perfect LD (r^2^ = 1, D’ = 1) and had the strongest nominal associations with TMT B scores (p_raw_ = 0.002). **TMT B-A**. For the score difference between TMT B and TMT A, nine variants were identified to be significantly associated with these scores. The strongest association was found at rs75011399 (p_raw_ = 3.4e-05, p_adj._ = 0.0087). Another variant, rs537022445, was also significantly associated with TMT B-A after the Bonferroni correction (p_raw_ = 0.00017, p_adj._ = 0.044). This SNP was found in perfect LD with rs543000211 (r^2^ = 1, D’ = 1). More information about significantly associated hits with cognitive functions can be found in Table 2.

**Table 2:** Description of significant SNPs in genotype-cognition association analyses.

Cognitive test	SNP	Major/minor allele	MAF	Unadj. p-value**
Memory	rs185882149	A/G	0.00025	0.043
	rs78795600	A/G	0.027	0.017
	rs189010729	A/T	0.013	0.047
	rs533990845	T/C	0.00054	0.019
	rs10484629*	A/C	0.116	0.021
	rs11962883*	A/C	0.118	0.016
	rs6910639	A/G	0.118	0.021
TMT A	rs531880979	C/A	0.00031	0.030
	rs548918202	T/C	0.00019	0.037
TMT B	rs6917833	T/C	0.45	0.026
	rs75011399	C/T	0.00026	0.0041
	rs9399042	C/A	0.00066	0.022
	rs551529344	T/G	0.00070	0.041
	rs537022445*	C/T	0.00013	0.0022
	rs543000211*	G/A	0.00013	0.0022
TMT B-A	rs539782560	C/G	0.00057	0.0045
	rs537869171	A/G	0.00030	0.030
	rs78795600	A/G	0.033	0.035
	rs75011399	C/T	0.027	**3.40E-05**
	rs550261940	T/G	0.00026	0.0097
	rs527282915	C/T	0.00028	0.026
	rs555565026	A/G	0.32	0.0088
	rs551529344	T/G	0.00015	0.0054
	rs537022445*	C/T	0.00057	**0.00017**
	rs543000211*	G/A	0.00070	**0.00017**

* SNPs in LD (r^2^>0.8, D’>0.8)

**Genotype-cognitive phenotype association analyses were performed, after adjusting for age, sex, education, assessment centre, genotyping batch, genotyping array and 10 principal components

Unadj.p-values that passed the Bonferroni correction are written in bold

Abbreviations: SNP, single nucleotide polymorphism; MAF, minor allele frequency; TMT, trail making test

## Discussion

The SLC18B1 gene is coding for a solute carrier that is most similar to vesicular transporters transporting monoamines and acetylcholine. Recently it has been suggested, through *in vitro* experiments on synthetic liposomes, that this transporter transports polyamines [10] and thus being the only known transporter in mammals with the ability to transport polyamines. Our results show a significant (P = 0.011) reduction in polyamine content in the brain of cKO mice (Fig 1F) as compared to ctrl mice. This could suggest that *Slc18b1* is also expressed in the plasma membrane of neurons and have a role in supplying the neurons with polyamines. It is also possible that reduced levels of polyamines observed in the cKO compared to ctrl mice could be a secondary effect of removal of the vesicular expression of *Slc18b1* altering the homeostasis of polyamines in the brain. Our data thus confirm and strengthens previously published data that *Slc18b1* is indeed able to mediate transport of polyamines and most importantly we here show that this is also its physiological role *in vivo*.

Thorough behavioural characterization of the cKO mouse revealed no major phenotype on basic behavioural such as anxiety, depression or locomotion. However, we found a strong phenotype with robust effects on both short and long term memory. In the radial arm maze, where the cKO mice perform significantly worse, displaying impaired learning ability (p<0.0001) as well as impaired working memory (p<0.0065) compared to controls. Also, the cKO mice made more reference memory errors by having significantly more visits into never baited arms (p = 0.037). Moreover, the novel object recognition displayed that the cKO mice had both poorer short-term memory (P< 0.05) as well as long-term memory (P<0.05) by spending less time with the novel object on both testing days. Our results regarding the memory phenotype resembles those of knockout studies of several proteins in the LTP pathway. For example, loss of function mutations of CamKII, a key molecule in the early phase of LTP [24], results in impaired spatial memory in the Barnes maze, similar to the Slc18b1 KO mice [25]. Also, CamKII heterozygote null mice show phenotype in memory similar to Slc8b1 KO mice, although the magnitude of the effect presented is not as strong as the effect we present here [26]. Interestingly, we see a significant reduction of CamKII in cKO compared to ctrl mice at the protein level (Fig 5), supporting the notion of the involvement of Slc18b1 in the LTP pathway. In addition, loss of function mutations or pharmacological inhibition of map kinases acting downstream of CamKII such as MEK1 [27] and ERK1/2 [28] shows impaired hippocampal function in the contextual fear conditioning paradigm. These results could be considered similar to the results we obtain in the novel object recognition, since both paradigms measure declarative memory at the hippocampal level [29].

We also assessed the voluntary consumption of high-sucrose food in an operant self-administration paradigm [30]. We could not detect any deficiencies in the reinforcing properties of sugar as cKO and ctrl mice responded equally in the FR5 as well as in the PR paradigm. We found however that the cKO mice performed worse during the training as well as the reinstatement phase, indicating that they do not comprehend the task as fast as ctrl mice, again pointing toward impaired memory formation. The hippocampus is a region involved in processing of declarative and contextual information [31] which is used during the SA paradigm. This again points towards impaired memory functions and reduced LTP in the cKO mice, corroborating our data from the radial arm maze and novel object recognition paradigms.

In order to know more about the molecular characteristics of the knockout, we performed both transcriptomic and proteomic analysis. We found no clustering by genotype based on all transcripts with detected expression (28794 transcripts). However, when we used the 500 transcripts with lowest P-value for differential expression, we found a clear clustering on based on genotype. Interestingly, the follow up pathway analysis suggests effects on systems related to memory and plasticity (“Huntington’s disease” and “Alzheimer’s diseases”), phosphorylation (“oxidative phosphorylation”) and receptor ligand interactions (“Neuroactive ligand–receptor interaction”). These pathways all have GABA and glutamate signalling as important components, which prompted us to investigate in detail effects on these systems.

We used GSE (Gene Set Enrichment Analysis) on seven sets of genes; all related to GABA or glutamate signalling and found interesting patterns. The NMDA and AMPA receptor subunits which represent the postsynaptic ionotropic glutamate receptors were downregulated. A downregulation of these systems would result in a lowered postsynaptic response from glutamate. The extrasynaptic ionotropic glutamate receptors of the kainite (GIRK) family had an enrichment score with a trend (p = 0.06) towards significant upregulation in the cKO mice. We also observe a pattern with the metabotropic glutamate receptors (mGluRs). These are G protein-coupled receptors and are divided into three groups (I-III). Of these mGluR1 and mGluR5 constitutes group I, being stimulatory receptors and both these are positively coupled to calcium, and are mainly postsynaptic [32, 33]. We found that both group I receptors are strongly downregulated in the knockout mice which would give a reduced response to glutamate in the postsynaptic neuron, in line with the results found for NMDA and AMPA receptors. Interestingly also *mGlur7*, a member of the group III mGluRs was also strongly downregulated in the cKO mice. This is the main pre-synaptic receptor and is negatively coupled to cAMP and a downregulation of this protein would result in a lowered negative feedback from glutamate and therefore more glutamate release, which is in line with the results from the kainite receptors. Taken together, we see a marked downregulation of postsynaptic glutamate receptors and an increase of expression in extrasynaptic and presynaptic glutamate receptors. The remaining pathways, except “steroid hormone metabolism”, we identified were related to immune function. This could be a direct effect of reduced release of spermidine and other polyamines, because theseare known to reduce immune responses, in particular to regulate division and differentiation of immune cells [34]. Recently it was also showed that mast cells have the capability to release the polyamines spermine and spermidine. Mast cells are secretory cells that play an important role in host defence [35]. However, how this relates to immune function genes in the brain, the tissue used in our study, is unclear and has not been investigated.

We also analysed differences in the global proteome using mass-spectrometry and identified 34 proteins that were significantly altered between cKO and ctrl mice. These pathways corroborated well with those that were significantly altered at the transcriptome level. Apart from the immune system related pathways, we identified effects on “Long Term Potentiation”, “SNARE interactions in vesicular transport” and “calcium signalling”. Among those proteins that were significantly changed (Table 3), we found altered levels of CamKII, which is a key molecule in the LTP pathway [36] as well as synaptic proteins Vamp2, Slc6A17 and Rab14 [37–39]. We also found changes in several β-Tubulins, suggesting changes in neuronal structure and morphology [40].

**Table 3:** Fraction 1 indicates hydrophobic fraction and fraction 2 indicates hydrophilic fraction.

UniProt Symbol	Protein name	UniProt ID	P-value (Direction)	Fraction
2AbA	Serine/threonine-protein phosphatase 2A 55 kDa regulatory subunit B alpha isoform	Q6P1F6	0.042(+)	2
Aqp4	Aquaporin-4	P55088	0.022(-)	1
Atp1a3	Sodium/potassium-transporting ATPase subunit alpha-3	Q6PIC6	0.049(+)	2
Atp6v1b2	V-type proton ATPase subunit B	P62814	0.012(-)	2
Atp6v1c1	Vacuolar proton pump subunit C 1	Q9Z1G3	0.042(-)	2
Camk2a	Calcium/calmodulin-dependent protein kinase type II	P11798	0.029(-)	1
Caza1	F-actin-capping protein subunit alpha-1	P47753	0.012(-)	2
Dhpr	dihydropteridine reductase	Q8BVI4	0.003 (+)	2
Dnm3	Dynamin-3	Q8BZ98	0.031(-)	2
Ef1a1	Elongation factor 1-alpha 1	P10126	0.046(+)	2
Ezr	Ezrin	P26040	0.031(+)	2
Gpil	Glucose-6-phosphate isomerase	P06745	0.031(+)	2
Ndus1	NADH-ubiquinone oxidoreductase 75 kDa subunit	Q91VD9	0.034(-)	2
Ndrg1	Protein NDRG1	Q62433	0.018(-)	2
Nfasc	Neurofascin	Q810U3	0.021(+)	2
Nrcam	Neuronal cell adhesion molecule	Q810U4	0.032(-)	2
Pak1	Serine/threonine-protein kinase PAK 1	O88643	0.023(-)	2
Pip4k2a	Phosphatidylinositol 5-phosphate 4-kinase alpha type-2	O70172	0.020(-)	2
Pp2b2	Serine/threonine-protein phosphatase 2B catalytic subunit beta isoform	P48453	0.026(-)	2
Rab14	Ras-related protein Rab-14	Q91V41	0.012 (-)	1
Ran	GTP-binding nuclear protein Ran	P62827	0.028 (-)	2
Rap1a	Ras-related protein Rap-1A	P62835	0.0035(-)	1
Rhoa	Transforming protein RhoA	Q9QUI0	0.012(+)	1
Rs18	40S ribosomal protein S18	P62270	0.036(+)	1
Sept3	neuronal-specific septin-3	Q9Z1S5	0.008 (+)	2
Slc6a17	Sodium-dependent neurotransmitter transporter NTT4	Q8BJI1	0.038(+)	1
SSDH	Succinate-semialdehyde dehydrogenase	Q8BWF0	0.020(-)	2
Tcpq1	T-complex protein 1 subunit theta	P42932	0.003(+)	2
Tuba1a	tubulin alpha-1A chain	P68369	0.009(+)	2
Tubb2b	Tubulin beta-2B chain	Q9CWF2	0.030(-)	2
Tuba4a	tubulin alpha-4A chain	P68368	0.004(+)/0.007(+)	1/2
Tubb5	tubulin beta-5 chain	P99024	0.006(+)	2
Ube2v2	Ubiquitin-conjugating enzyme E2 variant 2	Q9D2M8	0.022(+)	2
Vamp2	Vesicle-associated membrane protein 2	P63044	0.004(-)	1

In order to further investigate the role of GABA and glutamate signalling pathways, we performed additional behavioural experiments. We injected the animals with diazepam, a positive steric modulator of the GABA_A_ receptor that enhances the affinity of endogenous GABA [41]. We find a remarkable difference in the total activity after administration of diazepam. The cKO mice only reduced their activity with approximately 30% while the ctrl mice reduced their activity approximately threefold. The reduced response to diazepam could be the result of a reduced GABA signalling ability of the cKO line due to a long term reduction in glutamate tonus. This would result in a lower effect of diazepam that needs GABA to be active. We also administered the stimulatory drug amphetamine which is known to enhance glutamate levels [42] and increases excitatory glutamatergic signalling [43]. Our results shows a higher increase in total activity in the cKO mice compared to ctrl mice, which is also consistent with reduced GABA activity, i.e. a less strong inhibition on the nervous system. The reduction of GABA activity could be either at the receptor level, which is indicated by our micro array analysis data, or at the amount of GABA itself. However, when we measure amount of GABA in total brain of the knockouts, we see no differences between genotypes which is also true for glutamate (Fig 2E and 2F). This suggests that the changes in the GABA system are at the receptor level rather than at the transmitter level. It should however be noted that our measurements of GABA and glutamate is in whole brain extract and there could still be differences regarding the partition of the transmitters, for example amount of extracellular versus intracellular, or the amount packed into synaptic vesicles.

The robust effects on memory that we see where the cKO mice perform significantly worse than controls are in good agreement with the proteomics results on LTP. This could be a result of reduced NMDA receptor signalling. The NMDA receptor is crucial for formation of LTP and memory ([44] and polyamines, especially spermidine, has been shown to strengthen glutamatergic signalling through the NMDA receptor [45] in neuronal cultures and that this could have an effect of LTP [36] and hence neuronal plasticity. We do show (Fig 1F) that cKO mice have significantly lower polyamine content in neurons and we also show that NMDA receptor transcripts are downregulated. Taken together our data show that cKO mice have reduced levels of several molecules involved in LTP formation, including the NMDA receptor, CamKII and spermidine and that lack of the polyamine transporter *Slc18b1* results in these changes. Our current data do not identify which of the polyamine species are changed, which is a limitation of the present study, as we measure total polyamines (Fig 1F). It would be of interest to perform a thorough analyse of the entire polyamine system, including synthesis enzymes, precursors and metabolites, to fully understand the impact on the polyamine system from removal of Slc18b1. It would also be of high interest to understand in which subcellular compartments these changes have occurred. Our data do however show that there are effects on levels of polyamines in total from removing the presumably mainly vesicularly expressed Slc18b1, which is in itself an interesting finding. In addition, lower GABA levels have been shown to affect memory functions [46, 47] and especially the GABA_A_ is linked to memory formation [46]. Our expression analysis shows downregulation of several GABA-A receptor subunits (Fig 5c) and our pharmacological treatment with diazepam (Fig 6A) also points towards a reduced function in the GABA system. Most likely, the memory impairments in the cKO mice are results of dysfunction in both the GABA and glutamate systems.

To conclude we show that targeted deletion of *Slc18b1* generating null KO mice with reduced polyamine content in neurons. Deletion of *Slc18b1* also results in impaired memory functions, profoundly altered expression of genes involved in LTP, plasticity, calcium signalling and synaptic function. We discuss potential effects on the GABA and glutamate system based on the transcriptomics and proteomics data that are well corroborating that the mouse has reduced response to the GABA enhancing drug diazepam.

## Materials and methods

### Ethics statement

All animal procedures followed Swedish (Animal Welfare act) regulation and European Communities Council Directive (86/609/EEC) and were approved by the Uppsala Ethical committee for use of animal.

### Transgenic mice

#### Generation of transgenic mice

We generated the transgenic SLC18B1 allele by replacing part of the SLC18B1 gene with a targeting construct by homologous recombination in ES cells (Fig 1A). Successfully targeted ES from SV/129 cells produced a recombinant allele with two lox p sites floxing exon 3, 4 and 5 on either side followed by neomycin cassette enclosed within the frt sites which were screened using a combined southern blotting and PCR strategy. Two positive clones of ES cells were selected for injection into the blastocyst and further transferred into foster mother of C57BL/6 to generate chimeric mice. These were bred with C57BL/6 mice to generate heterozygous mice carrying one floxed allele, *Slc18b1*^*f*/+^. These mice were intercrossed to produce homozygous “floxed” mice *Slc18b1*^*f/f*^. Deleter-FlpE mice [15] was crossed with *Slc18b1*^*f/f*^ mice to remove the neomycin cassette and the “flipped” *Slc18b1*^*f/f*^ mice were viable and fertile. The “flipped” *Slc18b1*^*f/f*^ mice were crossed to PGK-Cre [16] mice to generate null mutants *Slc18b1*^*f/f;PGK-Cre*^ conditional KO (cKO) mice.

#### Genotyping

Tail biopsies (1–2 mm) were incubated in 75 ul of Buffer I consisting of 25 mM NaOh and 200 uM ethylenediaminetetraacetic acid (EDTA) at 95°C for 45 min and placed on ice for 10 min before adding 75 ul of Buffer II consisting of Tris-HCl (40 mM), pH 8.0. Mice were genotyped for the presence of the floxed alles and the Cre recombinase, using primers; P1: (ctg aga agc agg ctc agg tt), P2: (ggg tac cga gct cga att act) and P3 (tcc aac cac cca agt agt gg). In addition, Neo and Deleter-FlpE specific PCRs were used to genotype Neo-excised mice.

#### Verification of Slc18b1 loss through Western blotting

A ctrl male and a cKO male mouse were sacrificed by cervical dislocation and the brains were dissected and divided into smaller pieces. All chemicals were purchased from Sigma-Aldrich, USA unless otherwise stated. 1 tablet protease inhibitor cocktail (Roche Diagnostics, Sweden) was dissolved in 50 ml PBS (137 mM NaCl, 2.7 mM KCl and 10 mM Na_2_HPO_4_, pH 7.4) and 5 volumes of PBS/ inhibitor mix were added to the brains and the brains were homogenized in Dounce homogenizer with 25 strokes. The proteins were centrifuged for 10 min at 17000rpm. The supernatants were removed and the pellets were dissolved in 5 ml PBS/inhibitor and the membranes containing membrane protiens were collected by centrifugation at 1000 x g for 5 minutes. The supernatants (S_0_) were removed and the pellets were dissolved in 1 ml homogenization buffer (50mM Tris, 150mM NaCl, 4mM MgCl, 0.5mM EDTA, 2% Triton-X and 1 protease inhibitor cocktail tablet /50 ml buffer). The dissolved pellets were centrifuged at 10000 x g for 10 min and the supernatants (S_1_) were transferred and to a new Eppendorf tube. The pellets (P_1_) were dissolved in 200μl homogenization buffer. The supernatants (S_1_) were centrifuged at 15000g for 15 min and the supernatants (S_2_) were transferred to a new tube and the pellets (P_2_) were dissolved in 200μl homogenization buffer. The protein concentrations were measured with D_C_ protein assay kit (Bio-Rad, USA) following the manufactures protocol in 96 well BRANDplates pureGrade^™^ (BRAND GMBH, Germany). 50μg of proteins from all fractions were diluted in MQ water to a total volume of 15μl and 10μl of sample buffer (95% Lammeli’s sample buffer (Bio-Rad, USA), 5% 2-mercaptoethanol (Fluka, USA)) were added and the samples were incubated at 95°C for 5 minutes. 25μl of samples were loaded in wells together with 5μl Page ruler Prestained ladder, 250μg (Fermentas, Sweden). Electrophoresis were performed at 150V for 30 minutes with gel Mini-protean TGX Precast Gels 4–15%, 10 well comb, 50 μl/well (Bio-Rad, USA) with running buffer (0.025 M Trizma base, 0.192 M Glycine, 0.1% SDS. The proteins were transferred to an immobilian Transfer Membrane (PVDF, 0.45 μm, Millipore, USA) in transfer buffer (0.025M Trizma base, 0.192M Glycine, 20% methanol). The membrane was blocked in blocking buffer (5% Blotting grade blocker Non-fat dry Milk (Bio-Rad, USA) in TTBS (0.15M NaCl, 0.01M Trizma base, 0.05% Tween-20, pH = 8.0)) for 1 hour and incubated in anti-SLC18B1 antibody (AV50202, Sigma-Aldrich, USA) diluted 1:1000 in blocking buffer overnight at 4°C. The membrane was washed 3*10 minutes in TTBS before and after incubation in goat-anti-rabbit horseradish peroxidase antibody (Invitrogen, USA) diluted 1:10000 in blocking buffer for 1 hour. The membrane was incubated in developing mix 1:1 of luminol/enhancer and peroxidase buffer solution (Immune- Star HRP, Bio-Rad, USA) for 3 minutes and developed on Amersham Hyper film ECi, high performance chemiluminescence (GE Healthcare, USA) for 10 minutes. The membrane was washed in TTBS 2*30 min before and 3 *10 min after incubation in anti-mouse β-actin (Sigma, A1978) diluted 1:5000 in blocking buffer for 1hour. The membrane was incubated in goat- anti-mouse horseradish peroxidase antibody (Invitrogen, USA) diluted 1:10000 in blocking buffer and washed 3*10 minutes in TTBS and developed as earlier described for 5 minutes.

### Measurements of total polyamine content in brain

Brains from 10 week old cKO and ctrl mice (n = 7 ctrl, n = 7 cKO) were collected and stored in -80°C until the run of the experiment.

The polyamine measurement was performed with the flourometric Total Polyamine Assay Kit (Cat. nr: K475-100, BioVision Incorporated, CA, USA). Briefly, the brain was sagittal cut along the middle and homogenized in Polyamine Assay Buffer in a Bullet blender (Next advance, USA). A sample Clean-Up mix was used and sample spun in a 10kDa Spin Column (BioVision Incorporated). Samples were run in triplicates with one background control per sample. A standard curve was used to calculate the amount of polyamines in the samples.

### Quantification of transmitters using NMR

#### Sample preparation for NMR

For targeted NMR-based metabolomics analysis, brain samples were prepared and measured using methods previously described after slight modification [48]. Frozen brain samples (100 mg) were homogenized (Ultraturax T25, IKA, Staufen, Germany) in ice-cold methanol/chloroform (2:1, v/v, 3 mL) for 1 min and then sonicated in an ice-cold water bath for 30 min. After addition of 1 mL of ice-cold water and 1 mL of ice-cold chloroform, samples were centrifuged (1800g, 4 °C) for 35 min to achieve phase separation. The aqueous supernatant was collected, dried using an evacuated centrifuge (Savant, SVC 100H, Techtum Instrument AB, Umeaå, Sweden), and re-dissolved in 520 μL of sodium phosphate buffer (0.135 mol/L, pH 7.0). The residual proteins were then removed using Nanosep centrifugal filters (3 kDa, Pall Life Science, Port Washington, USA). The filtrate (390 μL) was mixed with extra phosphate buffer (130 μL, 0.135 mol/L, pH 7.0), D2O (50 μL), and sodium-3- (trimethylsilyl)-2,2,3,3-tetradeuteriopropionate solution (TSP-d4, 30 μL, 0.3 mmol/L, Cambridge Isotope Laboratories, Andover, USA). For NMR analysis 580 μL of mixture was added to 5 mm NMR tubes.

#### NMR analysis

The samples were analyzed by a 600 MHz Bruker NMR spectrometer using zgesgp pulse sequence (Bruker Spectrospin Ltd., BioSpin, Karlsruhe, Germany) at 25 °C with 128 scans. ^1^H NMR spectra were recorded with 65 536 data points over a spectral width of 17 942.58 Hz. The acquisition time was 1.8 s and the relaxation delay 4.0 s. All NMR spectra were processed using Bruker TopSpin 3.1 software. The data were Fourier-transformed after multiplication by a line broadening of 0.3 Hz and referenced to internal standard peak TSP-d4 at 0.0 ppm. For each spectrum, baseline and phase were corrected manually. Fourty-two metabolites were identified according to the NMR Suite 6.1 library (ChenomX Inc., Edmonton, AB, Canada), the Human Metabolome Database [49], and previous literature [50]. Concentrations of metabolites were calculated from the NMR spectra after accounting for interfering signals using NMR Suite 6.1 profiler as previously described [50] and expressed in μmol/g.

### Microarray expression analysis

#### Affymetrix microarray procedure

5 seven week old male cKO and 5 ctrl litter mates were sacrificed by cervical dislocation and the brains were dissected. The region between bregma 3 and -5 was used for analysis and was divided into two halves along the midline. One half was used for the micro array analysis and the other half for proteomics analysis (see below). The tissue was divided into smaller pieces (approximately 2mm^3^) and immersed in RNAlater solution (Ambion, USA) for 2 hours at 4°C and subsequently stored at -20°C. Total RNA was extracted using RNeasy mini kit (Qiagen, Netherlands), the RNA concentration was measured with a NanoDrop ND-1000 spectrophotometer (NanoDrop Technologies, USA). The Agilent 2100 Bioanalyzer system (Agilent Technologies, USA) was used for evaluation of RNA quality. From each sample a total of 250 ng of RNA was used to generate amplified and biotinylated sense-strand cDNA from the entire expressed RNA pool according to the Ambion WT Expression Kit (P/N 4425209 Rev B 05/2009) and Affymetrix GeneChip WT Terminal Labelling and Hybridization User Manual (Affymetrix, USA) (P/N 702808 Rev. 1). GeneChip ST Arrays (GeneChip Mouse Gene 1.0 ST Array) were hybridized for 16 hours, rotated at 60 rpm, at 45°C. According to the GeneChip Expression Wash, Stain and Scan Manual (Affymetrix, USA) (PN 702731 Rev 2) the arrays were then washed and stained using the Fluidics Station 450 and finally scanned using the GeneChip Scanner 3000 7G.

#### Microarray data analysis

The raw data were normalized using the robust multi-array average (RMA) method [51] using the Affymetrix Expression Console software. Thereafter analysis of the gene expression data was carried out in the freely available statistical computing language R (http://www.r-project.org) using packages available from the Bioconductor project (www.bioconductor.org). An empirical Bayes moderated t-test [52] was applied by using the ‘limma’ package [53]to search for the differentially expressed genes between the cKO and the ctrl samples. To control false discovery rate, the p-values were adjusted using the method of [37]. To study if the mice cluster by genotype a three dimensional principal component analysis (PCA) was performed in MATLAB (Mathworks, USA). Further a PCA plot in two dimensions including the 500 genes with lowest P-value (FDR corrected) for differential expression the two lines was performed.

### Proteomics analysis

#### Brain tissue

Brain tissue from cKO and ctrl mice was dissected as describe in the micro array analysis section, one half of the brain was used for microarray analysis and the other half for proteomics analysis.

#### Chemicals and reagents

Acetonitrile (ACN), methanol (MeOH), acetic acid (HAc), formic acid (FA), ammonium bicarbonate (NH_4_HCO_3_), tri-n-butylphosphate (TBP), sodium chloride (NaCl) were obtained from Merck (Darmstadt, Germany). Acetone, ethylenediaminetetraacetic acid tetrasodium salt dihydrate (EDTA), protease inhibitor cocktail, phosphate buffered saline (PBS), Tris-HCl, diethylamide (DEA), trifluoroacetic acid (TFA), n-octyl-β-D-glucopyranoside, triethyl ammonium bicarbonate (TEAB), and formaldehyde CH_2_O (37% (vol/vol)) were purchased from Sigma Aldrich (St. Louis, MO, USA). For tryptic digestion, iodoacetamide (IAA), urea and dithiothreitol (DTT) were obtained from Sigma Aldrich and trypsin (sequencing grade from bovine pancreas 1418475; Roche diagnostic, Basel, Switzerland) were used. Formaldehyde (^13^CD_2_O) (20% (vol/vol), 99% 13C, 98% D) and sodium cyanoborodeuteride (NaBD_3_CN) (96% D) were purchased from Isotec (Miamisburg, OH). Sodium cyanoborohydride (NaBH_3_CN) was obtained from Fluka (Buchs, Switzerland). Sucrose was purchased from Fisher Scientific Company (Göteborg, Sweden). Triton X-114 was obtained from KEBO Lab (Stockholm, Sweden). Ultrapure water was prepared by Milli-Q water purification system (Millipore, Bedford, MA, USA).

#### Cloud point extraction of proteins

Commercially available Triton X-114 was precondensated to obtain a homogenous Triton X-114 mixture [54]. Aliquots of 50 mg brain powder were homogenized for 60 seconds in a blender (POLYTRON PT 1200, Kinematica) with 1 mL of Triton lysis buffer (1% (v/v) Triton X-114, 10 mM Tris-HCl pH 7.4, 0.15 M NaCl, 1mM EDTA). Protease Inhibitor Cocktail (10 μL) was added during the sample preparation to prevent protein degradation. After homogenization, the sample was incubated for 1 hour at 4 °C during mild agitation. The cell lysate was clarified by centrifugation for 30 min (10000 × g at 4 °C) using a Sigma 2K15 ultracentrifuge (Sigma Laborcentrifugen GmbH, Osterode, Germany). The clear supernatant was then transferred directly onto 100 μL of sucrose cushion buffer and incubated at 37 °C for 5 minutes, which lead to the clouding of the solution. The sample was centrifuged for 3 minutes (400 × g at 37 °C) to separate the two phases; aqueous on the top and detergent at the bottom. The aqueous phase was transferred to a new tube and incubated on ice. The detergent phase was mixed with 500 μL of cold PBS and phase separation was repeated again. The second detergent depleted aqueous phase was then pooled with the first and kept on ice. The detergent-rich fraction, containing hydrophobic membrane proteins, was mixed with 1.5 mL of cold PBS. The pool of detergent-depleted aqueous phase was re-extracted by adding of 50 μL of 11.4% Triton X-114 stock solution, incubated at 37 °C for 3 minutes and centrifuged for 3 minutes (400 × g at 37 °C). This aqueous phase contained hydrophilic water-soluble proteins.

#### Delipidation and protein precipitation

A delipidation protocol according to Mastro et al. was used [55]. Aliquots (100 μL) of the detergent-depleted aqueous and detergent-rich phases were mixed with 1.4 mL of ice-cold tri-*n*-butylphosphate: acetone: methanol mixture (1:12:1) and incubated at 4 °C for 90 min. The precipitate was pelleted by centrifugation for 15 min (2800 × g at 4 °C) and then washed sequentially with 1 mL of acetone and 1 mL of methanol, and finally air dried.

#### Protein quantification

The total protein content of delipidated proteins was determined using the DC Protein Assay Kit (BioRad Laboratories, Hercules, CA, USA), which is based on the modified Lowry method with bovine serum albumin as standard [56]. The protein pellets were dissolved in 100 μL of 6% SDS. The DC assay was carried out according to the manufacturer’s instructions using 96-well microtiter plate reader model 680 (BioRad Laboratories).

#### On-filter tryptic digestion of proteins

The delipidated samples were redissolved in 100 μL of 50:50 ACN: 8M urea + 1% n-octyl-β-D-glucopyranoside. Aliquots corresponding to 20 μg of proteins were taken for digestion. An on-filter digestion protocol was used for tryptic digestion of the samples [57] using 3 kDa filters (Pall Life Sciences, Ann Arbor, MI, USA). Centrifugation was carried out at a centrifugal force of 14,000xg throughout the protocol. The samples were first redissolved in 100 μL of 50:50 ACN: 8M urea + 1% n-octyl-β-D-glucopyranoside. A volume of 10 μL of 45 mM aqueous DTT was added to all samples and the mixtures were incubated at 50 °C for 15 min to reduce the disulfide bridges. The samples were cooled down to room temperature and 10 μL of 100 mM aqueous IAA was added and the mixtures were incubated for an additional 15 min at room temperature in darkness to carabamidomethylate the cysteines. The samples were transferred to spin filters that had been pre-washed with 250 μL of 50% ACN for 15 min and then 500 μL of water for 20 min followed by centrifuged for 10 min to remove the added salts, detergents and other interfering substances. An additional volume of 100 μL of 2% ACN in 100 mM TEAB was added and the filters were spun for 10 min followed by 100 μL of 50:50 ACN: 100 mM TEAB and 100 μL of 100 mM TEAB, and centrifugation for another 10 min. Finally, a volume of 100 μL of 50 mM TEAB was added together with trypsin to yield a final trypsin/protein concentration of 2.5% (w/w). The tryptic digestion was performed at 37 C overnight in darkness. Samples were subsequently centrifuged for 20 min to collect the tryptic peptides in the filtrate while retaining undigested proteins and trypsin in the retentate. An additional volume of 100 μL of 50% ACN, 1% HAc was added and the filters were spun for 10 min and pooled with the first tryptic peptide filtrate. The collected filtrates were vacuum centrifuged to dryness using a Speedvac system ISS110 (Thermo Scientific, Waltham, MA, USA).

#### Stable-isotope dimethyl labeling

The peptides resulting from the on-filter tryptic digestion of cKO and ctrl samples were isotopically labeled using reductive dimethylation according to [58] with light and heavy label, respectively. The peptide mixture was dissolved in 100 μL of 100 mM TEAB. For the light and heavy labeling, 4 μL of CH_2_O (4%, v/v) and ^13^CD_2_O (4%, v/v) were added into the sample solution, respectively. The mixture was briefly vortexed and, then, 4 μL of freshly prepared 0.6 M NaBH_3_CN and 0.6 M NaBD_3_CN were added subsequently. The resultant mixture was incubated for 1 h at room temperature while mixing. Then, 16 μL of ammonia (1% in water) and 8 μL of formic acid were added to consume the excess labeling reagents and acidify for the subsequent solid phase extraction (SPE). Then two differentially labeled samples were pooled in a 1:1 ratio and the labeled peptide mixture was desalted by the SPE column.

#### Sample desalting

The labeled peptide mixtures were desalted on a Isolute C18(EC) (1 mL, 50 mg capacity, Biotage, Uppsla, Sweden) SPE column using the following schedule: The column was first wetted in 500 μL of 100% ACN and equilibrated with 5×500 μL 1% HAc. The tryptic peptides were adsorbed to the media using 5 repeated cycles of sample loading. The column was washed using 5×1 mL of 1% HAc and finally the peptides were eluted in 250 μL 50% ACN, 1% HAc. After desalting, the eluate was vacuum centrifuged to dryness.

#### NanoLC-MS/MS for protein identification

The protein nanoLC-MS/MS experiments were performed using a 7 T hybrid LTQ FT mass spectrometer (ThermoFisher Scientific, Bremen, Germany) fitted with a nano-electrospray ionization (ESI) ion source. On-line nanoLC separations were performed using a Agilent 1100 nanoflow system (Agilent Technologies, Waldbronn, Germany). The peptide separations were performed on in-house packed 15-cm fused silica emitters (75-μm inner diameter, 375-μm outer diameter). The emitters were packed with a methanol slurry of reversed-phase, fully end-capped Reprosil-Pur C_18_-AQ 3 μm resin (Dr. Maisch GmbH, Ammerbuch-Entringen, Germany) using a PC77 pressure injection cell (Next Advance, Averill Park, NY, USA). The injection volumes were 5 μL and corresponded to 2 μg of proteins. The separations were performed at a flow of 200 nL/min with mobile phases A (water with 0.5% acetic acid) and B (89.5% acetonitrile, 10% water, and 0.5% acetic acid). A 100-min gradient from 2% B to 50% B followed by a washing step with 98% B for 5 min was used. Mass spectrometric analyses were performed using unattended data-dependent acquisition mode, in which the mass spectrometer automatically switches between acquiring a high resolution survey mass spectrum in the FTMS (resolving power 50 000 FWHM) and consecutive low-resolution, collision-induced dissociation fragmentation of up to five of the most abundant ions in the ion trap. Acquired data (.RAW-files) were converted to the .mgf format using an in-house written program (C++) and subjected to protein identification using MASCOT search engine (version 2.2.2, Matrix Science, UK) against the SwissProt database version 51.6. The search parameters were set to Taxonomy: Mus musculus, Enzyme: Trypsin, Fixed modifications: Carbamidomethyl (C), Dimethyl (K, N-term), Dimethyl (D_6_^13^C_2_K, D_6_^13^C_2_N-term); Variable modifications: Oxidation (M) and Deamidated (NQ), Peptide tolerance: 10 ppm, MS/MS tolerance: 0.7 Da and maximum 2 missed cleavage sites.

#### Quantification

For data evaluation, the software MSQuant v2.05b5 was customized for duplex dimethylation and used for extraction and integration of ion chromatograms of all identified peptides. Low intensity peaks (S/N≤10) were excluded and the peak areas were automatically integrated. All peaks were visually inspected and corrected if needed. The MSQuant output was further used for calculation of median proteins ratios.

#### KEGG analysis

Significantly changed proteins were used in the KEGG pathway mapping tools (http://www.genome.jp/kegg/mapper.html) to identify pathways significantly enriched for proteins with significant difference between cKO and ctrl. The default settings for the multiple search object options were used.

### Behavioural analysis and animals

#### Animals

Mice were housed in constant temperature (21 ±1°C) and humidity (50–60%) with 2–8 mice per cage unless otherwise stated. All behavioural experiments took place during the light phase, between 09.00 and 17.00. Food (R3, Lactamin/Lantmännen/Sweden) and water was provided ad libitum unless otherwise stated. All behavioural test were conducted on adult male mice (>8 weeks). Control mice were litter mates control. All mice were maintained on the same genetic background, a combination of C57BlL6 and Sv129. The observer was blind to the genotype of the mice throughout the experimental periods.

#### Elevated plus maze

The elevated plus maze measure anxiety-like behaviour and consists of two open arms and two closed arm (40 cm high walls) situated 51 cm above the floor [59]. The mice (cKO male mice, n = 11; ctrl male mice, n = 9) was placed in the centre of the maze and the activity was videotaped for 10 min under dimmed lights, 4 lux in the closed arms and 10 lux in open arms. The latency, frequency, duration, head dips and rearing were scored. The observer scored the plus maze using the AniTracker Software (Fredriksson)Data was analysed using a non-parametric Mann-Whitney U test by the Prism Software (Graph pad). Values in graphs were expressed as mean ±SEM.

#### Rotarod

To assess the acquisition of motor skilled behaviour a rotarod machine with automatic timers and falling sensors were used (IITC Rotarod, Life Science) [60]. The mice (cKO male mice, n = 10; ctrl male mice, n = 10) were placed on textured drums (2, 8 cm in diameter) to avoid slipping. The rod was accelerating with the speed of 4 rpm to 40 rpm for two minutes. The mice went through three trials per day for three days. The mean for each day were analysed using a non-parametric Mann-Whitney U test by the Prism Software (Graph pad). Values in graph were expressed as mean ±SEM.

#### Long term food intake

12 cKO and 12 ctrl male mice were single housed in standard macrolon type III cages and kept under standard conditions with a 12/12h light/dark cycle (lights on at 0600) and tap water and high-fat/high-sugar diet (Brogaarden, Denmark) available ad libitum.

The animals entered the study at the age of 8 weeks and were kept on the high-fat/high-sugar diet for 13 weeks. Food intake and bodyweight were measured weekly. Data was analysed using a two- way ANOVA by the Prism Software (Graph pad). Values in graph were expressed as mean ±SEM.

#### Radial arm maze

Short term spatial memory was examined in a eight arm radial maze [61] (cKO male mice, n = 9; ctrl male mice, n = 11). The arm was 10 cm wide and 50 cm long with a central platform. At the distal end of each arm a recessed food plate was fixed at 1.5 cm above the maze floor. The same four arms were baited each time with a small piece of reinforcement pellet (5TUL, TestDiet; 50%kcal from sucrose). To increase motivation to find a reward, the mice were slightly food restricted by starving them overnight one night prior to the day of maze performance. The mice were placed in the centre of the maze to search for food. The mice were removed from the maze after they consumed all four pellets. Short- term spatial memory was analysed during a six day acquisition phase. Re-entry in to a previously baited (now empty arm) was defined as working memory error (WME). Entries in to never baited arms were defined as spatial reference memory error (RME). Data was analysed using a two- way ANOVA by the Prism Software (Graph pad). Values in graph were expressed as mean ±SEM.

#### Novel-object recognition test

The experimental arena consists of a round arena (Ø40 cm) with 40 cm high wall. The mice went through a three day protocol (n = 5/genotype). The first day the mice were subjected to a habituation session, mice were individually placed in the arena with two identical objects and were allowed to explore for 10 min. On day two one of the familiar objects were replaced with a novel object, which they were allowed to investigate and scored for 10 min investigating short-term memory. Long term memory was investigated on day three when one familiar object and one novel object was presented for the mice and they were allowed to interact freely with for 10 min. All trails were videotaped and scored with the automated software Ethovision XT 11.0 (Noldus, Netherlands). Exploration of an object was defined as touching, sniffing, climbing, or sitting on the object. Objects were always placed in the same location; but the location of the novel object relative to the familiar object was randomized for each test across mice. A total of 10 male cKO mice and 10 ctrl littermates were included in this study. Discrimination index between the novel and familiar objects were calculated as time spent with the familiar object subtracted from time spent with a novel object divided by the total amount of exploration with the novel and familiar object.

#### Diazepam challenge

Locomotor activity was measured by using an automated device consisting of a plastic cage (55x55x22) inside a ventilated and illuminated (10 lux) cabinet (Locobox, Kungsbacka Regerteknik AB, Sweden). Photo beams situated inside the box tracked the movement of the mice. On day one the mice (cKO male mice, n = 16; ctrl male mice, n = 9) were intraperitoneal (i.p.) injected with 10 ml/kg saline and were allowed to explore the cage for 60 min. 24 h later the mice were injected i.p. with 2 mg/kg Diazepam and put back in the same plastic cage for 60 min. The total activity was scored and data was analysed using a two- way ANOVA using the Prism Software (Graph Pad, USA). Values in the graph were expressed as mean ±SEM.

#### Amphetamine challenge

The same automated boxes were used as in the diazepam challenge. The first day the mice (n = 10/genotype) were allowed to explore the box for 60 min and thereafter returned to their home cage. 24 h later they were returned to the cage and the mice were injected with 10 ml/kg saline i.p. and put in the box for 60 min of monitoring. Thereafter the mice were subjected to amphetamine in four different doses i.p. injections 0,5 ml/kg, 1 ml/kg, 2 ml/kg and 4 ml/kg in ramdomized order, with a three day wash out period between each dose. Total activity was normalized against the saline values for each animal. Data was analysed using a two- way ANOVA with the Prism Software (Graph pad, USA). Values in the graph were expressed as mean ±SEM.

#### Operand self-administration

The mice (cKO male mice, n = 7; ctrl male mice, n = 8) were 7–8 weeks old and weighed 25.8±0.6 g at the start of the study. The mice were kept in standard Macrolon III cages in housing cabinets controlled for temperature (21–22 °C) and humidity (45–50%). A 12 h light/dark cycle was employed with lights on at 6 AM. Autoclaved rodent chow (R3, Lactamin/Lantmännen, Linköping, Sweden) was provided ad libitum unless otherwise stated. During the food restriction (initial training and first part of the operant study), 2.75 g (±10%) of rodent chow was provided each day at 6 PM.

For the initial training food-restricted mice were trained to nose poke for food pellets (5TUL, TestDiet; 50% kcal from sucrose) in operant chambers (MedAssociates). Behaviour was recorded via the MED-PC computer interface and MED-PC version 4 software control system. The chamber was equipped with a food receptacle for pellet deliveries with a nose poke response device on either side. The left nose poke hole was active throughout the study and responding at this manipulandum resulted, according to different schedules (see below), to the delivery of a food pellet coupled with the illumination of a stimulus light inside the nose poke hole and a stimulus sound (short burst of clicks). On the first day of training, both nose poke holes were active on a fixed-ratio-1 (FR1) schedule and behaviour was further shaped towards the food receptacle by the delivery of a free pellet every 2 min accompanied by the stimulus sound. During the initial training no more than 30 food pellets could be obtained in one session. The fixed ratio was gradually increased to FR5 (one day each of FR2 and FR3).

After six days of testing on the FR5 schedule, the mice were introduced to a progressive ratio schedule (PR) for three days, whereby the response requirement for each reward in a session increased according to the formula 5e^(reinforcement number × 0.2)^ − 5, rounded to the nearest integer [62]. After data were collected at the PR during food restriction, all mice were returned to ad-libitum home cage access to rodent chow. Next we wished to determine the animal’s propensity to self-administer sucrose pellets in a non-food restricted state and mice were evaluated at the FR5 schedule for 7 days and PR schedule for six days. Finally, we employed an extinction-reinstatement procedure whereby animals were first exposed to the operant chamber for six sessions during which responding at both nose poke apertures were without programmed consequence (extinction), thereafter the mice were returned to their home cage for five days. Thereafter the animals were tested on a single session with access to the operant chambers on a schedule in which responding at the active nose poke aperture produced the presentation of the stimulus light as well as the stimulus sound, but no pellet delivery (reinstatement). Data was analysed using a two- way ANOVA or Mann Whitney U test using the Prism Software (Graph pad). Values in the graphs were expressed as mean ±SEM. Outliers were removed using Grubbs outlier test.

### Analysis of human SNPs

#### Participants

UK Biobank project includes 502 549 participants that were recruited between 2006 and 2010. Baseline data of these participants were gathered at 1 of 22 assessment centers across England, Scotland and Wales. Self-reported questionnaires and physical measurements on cognitive functions, lifestyle, environmental and genetic data were available. For this study, 115 807 individuals (60 865 females, 54 942 males) aged 40–73 (mean 56.91 years, s.d. = 7.93) were analyzed, having genomic data at 72 355 667 imputed variants. More details about imputation could be found at the following URL (http://biobank.ctsu.ox.ac.uk/crystal/refer.cgi?id=157020). The present analysis was conducted under UK Biobank data application number 2348.

#### Cognitive tests

Memory was measured with the ‘pairs matching’ task on a touchscreen computer (#20132). Participants had to correctly match 12 ‘cards’ from a randomly arranged grid (see details at [63]). The investigated memory scores included the total number of errors made during this task, independent on the needed time. Prior analysis, they were log+1 transformed. In total, 35 715 participants that had memory scores were also genotyped.

Trail making test A and trail making test B (TMT A and TMT B) were introduced later, at followed-up time, between 2014 and 2015. For both tests, the total completing time (in seconds) was registered. No outliers were excluded for TMT A score analysis. Individuals had scored higher than 250 s for TMT B (n = 10) were excluded. The separate TMT A and TMT B scores were log-transformed. The untransformed score difference TMT B-TMT A (TMT B-A) was calculated in order to take into account the individual motor speed and visual search [64]. Participants with TMT B-A values <-50 and >150 were removed from further analysis. More details about TMT A and B in the UK Biobank could be found at [65]. A total of 31 408 genotyped participants were included for TMT A, 31 392 participants for TMT B and 31 340 for TMT B-A.

#### Genotyping and quality control

The first genotyping data release of UK Biobank included 152 249 individuals, that were genotyped using the UK BiLEVE array (n = 49 922) or the UK Biobak axiom array (n = 102 326). There is more than 95% overlap for these two arrays. A total of 33 batches were used for genotyping analysis. Quality control (QC) steps were performed at the Wellcome Trust Centre for Human Genetics and by Affymetrix (http://biobank.ctsu.ox.ac.uk/crystal/refer.cgi?id=155580) before data releasing.

Additional QC steps were applied for participants in this study. Individuals were excluded based on ethnicity (self-identified as ‘non-white British’ (#22006; n = 31 965)), QC failure in UK BiLEVE (#22050 and #22051; n = 385), genetic relatedness factor (#22012; n = 8 779), and gender mismatch (#22001; n = 0). A total of 115 807 participants were included in any further analysis.

#### Selection and QC of SNPs

A total of 4 138 variants were associated with *SLC18B1* gene in the Single Nucleotide Polymorphism database (dbSNP). The imputed file for chromosome six contained 832 variants. Based on Hardy-Weinberg equilibrium (p<1e-10) and minor allele frequency (MAF>0.0001), 256 SNPs were included in further analysis. Out of these 256 variants, fourteen were within 3’ or 5’ untranslated gene regions and seven within the coding region.

#### Statistical analyses

Hardy-Weinberg equilibrium (p<1e-10) and linkage disequilibrium (LD) (r^2^>0.8, D’>0.8) were assessed with PLINK [66]. Cognitive phenotypes analyses were adjusted for age, sex, education, assessment centre, genotyping batch, genotyping array and 10 principal components. Education was used as a binary variable indexing if the participant had or not attained a college or university-degree [63]. Genotype-phenotype associations were performed on the imputed file ‘chromosome 6’ using SNPTEST v.2.5.1 (https://mathgen.stats.ox.ac.uk/genetics_software/snptest/snptest.html). For the genotype dosage scores (method ‘expected’), an additive model was assumed. The results were corrected for multiple testing, using the Bonferroni correction. All two-tailed p-values <0.05 were considered significant.

## Supporting information

S1 Table Table containing all graphed data. (XLS)

S2 Table Table containing all significantly changed molecules identified in the proteomics analysis. (XLS)
